# Multifocal Autoimmune Pancreatitis Mimicking Pancreatic Neoplasm in a Young Adult: A Diagnostic Challenge

**DOI:** 10.7759/cureus.111229

**Published:** 2026-06-21

**Authors:** Sofia Prada, Maria Leonor Neves, Teresa Pereira, Marinela Major

**Affiliations:** 1 Medicine, Universidade de Lisboa, Lisbon, PRT; 2 Internal Medicine, Unidade Local de Saúde (ULS) Amadora/Sintra, Lisbon, PRT

**Keywords:** autoimmune pancreatitis, igg4, multifocal pancreatic lesions, pancreatic cancer mimic, pancreatic mass

## Abstract

Autoimmune pancreatitis (AIP) is an uncommon fibroinflammatory pancreatic disorder that may closely mimic pancreatic malignancy, particularly when presenting with focal or multifocal mass-like lesions. Prompt recognition is essential to prevent unnecessary invasive procedures, inappropriate oncological management, and to allow timely initiation of glucocorticoid therapy. We report the case of a 32-year-old call center employee with a history of asthma who presented with a three-week history of progressive epigastric pain radiating to the back. Notably, he had no clinical or biochemical evidence of obstructive jaundice, with a total bilirubin level of 0.24 mg/dL at presentation. Initial abdominal CT demonstrated multifocal poorly defined hypovascular pancreatic lesions associated with pancreatic enlargement, raising concern for a pancreatic neoplasm. Laboratory evaluation revealed preserved hepatic function, normal serum calcium and triglyceride concentrations, mild inflammatory response, elevated total immunoglobulin G (IgG) (1673 mg/dL), mildly elevated IgG4 (153.5 mg/dL), and positive antinuclear antibodies. Tumour markers were negative. Subsequent MRI showed multifocal enlargement involving the pancreatic head, body, and tail, with delayed homogeneous enhancement, preservation of the main pancreatic duct calibre, and peripheral capsule-like features. These findings strongly suggested autoimmune pancreatitis. Histological confirmation was not initially obtained because corticosteroid therapy had already been initiated following multidisciplinary evaluation. The patient demonstrated rapid clinical improvement and remained asymptomatic during corticosteroid tapering. Endoscopic ultrasound-guided tissue acquisition and urgent imaging reassessment were planned to consolidate diagnostic certainty and exclude alternative diagnoses. This case highlights the diagnostic complexity of autoimmune pancreatitis presenting as multifocal pancreatic masses and illustrates the challenges associated with initiating corticosteroid therapy before histological confirmation. Recognition of characteristic radiological patterns and integration of clinical and serological findings remain crucial to avoid unnecessary invasive or surgical interventions.

## Introduction

Autoimmune pancreatitis (AIP) is an uncommon form of chronic fibroinflammatory pancreatitis characterized by immune-mediated pancreatic injury and a typically favorable response to corticosteroid therapy [1]. AIP is currently classified into two clinicopathological subtypes: type 1 AIP, which represents the pancreatic manifestation of IgG4-related disease and is frequently associated with systemic involvement, and type 2 AIP, also referred to as idiopathic duct-centric pancreatitis, which is generally confined to the pancreas and often coexists with inflammatory bowel disease (IBD) [1-3].

Although AIP is rare, important epidemiological differences exist between its two subtypes. Type 1 AIP is more common, predominantly affects men, and typically presents in the sixth to seventh decades of life. In contrast, type 2 AIP tends to occur in younger individuals, usually in the fourth decade, without a marked sex predominance [1-3]. Recognition of these age-related patterns may assist in differentiating AIP from pancreatic adenocarcinoma, which generally occurs in older patients.

Despite increasing recognition, the diagnosis of AIP remains challenging because its clinical, serological, and radiological manifestations may closely resemble pancreatic malignancy [2,4]. Patients often present with non-specific abdominal symptoms and imaging findings such as focal or diffuse pancreatic enlargement, multifocal lesions, delayed enhancement, or mass-like appearances, frequently triggering extensive oncological investigation and, in some cases, unnecessary invasive procedures [4-6].

Current international diagnostic criteria recommend an integrated approach combining pancreatic imaging, serological markers, histopathological findings, evidence of other organ involvement, and response to corticosteroid therapy [1,7]. Nevertheless, in routine clinical practice, obtaining histological confirmation before treatment initiation may not always be feasible, particularly when clinical suspicion is high and early therapeutic intervention is considered necessary.

We report a case of probable AIP with imaging features favoring type 2 disease in a young adult initially investigated for pancreatic neoplasm, highlighting the diagnostic uncertainty and therapeutic challenges associated with initiating corticosteroid therapy before tissue confirmation.

## Case presentation

A 32-year-old man with a medical history of asthma presented with a three-week history of progressively worsening epigastric pain radiating to the back in a belt-like distribution. Symptoms initially improved with fasting but gradually intensified, ultimately leading to multiple emergency department visits. He denied fever, weight loss, night sweats, anorexia, symptoms suggestive of pancreatic exocrine insufficiency, including diarrhea or steatorrhea, arthralgia, mucocutaneous manifestations, recent medication changes, or other systemic symptoms. There was no history of regular alcohol consumption, although he reported a single episode of binge drinking shortly before symptom onset.

On admission, the patient was haemodynamically stable and appeared in good general condition. Physical examination was notable only for mild epigastric tenderness, without guarding, palpable masses, organomegaly, or scleral icterus.

Initial laboratory work-up showed preserved liver function, normal serum calcium and triglyceride levels, mild inflammatory marker elevation, and no significant pancreatic enzyme abnormalities. Tumour markers, including carcinoembryonic antigen (CEA) and carbohydrate antigen 19-9 (CA 19-9), were within normal limits. Immunological evaluation (Table 1) demonstrated elevated total IgG (1673 mg/dL), mildly increased IgG4 levels (153.5 mg/dL), and positive antinuclear antibodies (ANA) (nucleolar pattern, titre 1:160).

**Table 1:** Laboratory investigations during hospitalization ASTP: aspartate aminotransferase; ALT: alanine transaminase; GGT: gamma-glutamyl transferase; ANA: antinuclear antibody; CEA: carcinoembryonic antigen; CA: cancer antigen

Parameter (Reference Range)	Patient Value
Hemoglobin (13.0–17.0)	13.9 g/dL
Leukocytes (4.0–10.0)	9.3 ×10⁹/L
Platelets (150-410)	253 ×10⁹/L
C-reactive protein (CRP) (<0.50)	5.17 mg/dL (peak)
Amylase (28-100)	112.9 U/L (peak)
AST (<40)	20 U/L
ALT (<41)	18 U/L
Alkaline phosphatase (40-130)	89.2 U/L
GGT (<60)	28 U/L
Total bilirubin (≤1.20)	0.34 mg/dL
Creatinine (0.70-1.20)	0.94 mg/dL
Triglycerides (<165)	65 mg/dL
Total IgG (700-1600)	1673 mg/dL
IgG4 (<130)	153.5 mg/dL
IgA (70-400)	210.4 mg/dL
IgM (40-230)	64.9 mg/dL
ANA	Positive (1:160 nucleolar pattern)
CEA (<5.0)	2.81 ng/mL
CA 19-9 (<39)	<2.0 U/mL

Contrast-enhanced abdominal CT demonstrated pancreatic enlargement with multifocal ill-defined hypovascular lesions involving the pancreatic head, uncinate process, and tail, associated with mild peripancreatic inflammatory changes. Importantly, there was no significant dilatation of the main pancreatic duct. To further characterize the pancreatic abnormalities identified on CT and refine the differential diagnosis, pancreatic MRI with magnetic resonance cholangiopancreatography (MRCP) (Figures 1, 2) was performed, revealing multifocal enlargement involving the pancreatic head, body, and tail, with progressive delayed enhancement, preservation of the main pancreatic duct calibre, and peripheral capsule-like features.

**Figure 1:**
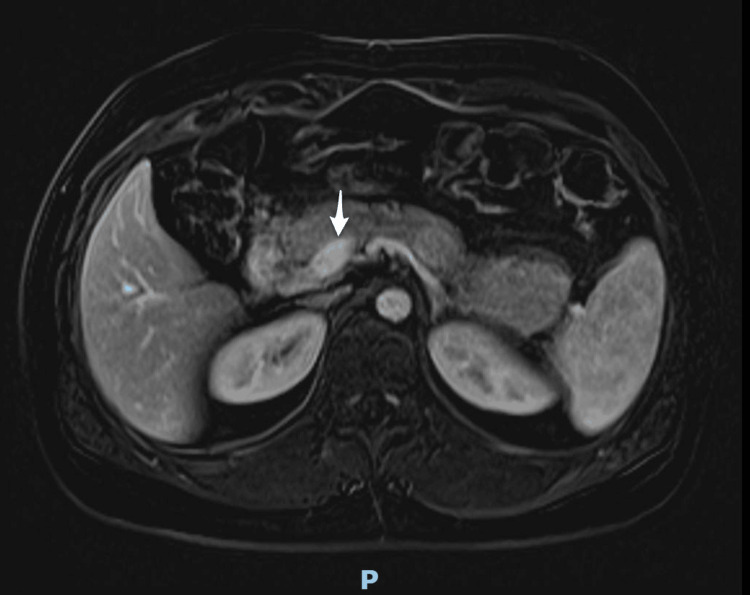
Early post-contrast axial pancreatic MRI demonstrating a focal hypovascular lesion involving the pancreatic body (white arrow). Additional pancreatic abnormalities were identified on complementary MRI sequences, supporting multifocal pancreatic involvement. The lesion demonstrates ill-defined margins and lacks features typically associated with pancreatic adenocarcinoma.

**Figure 2:**
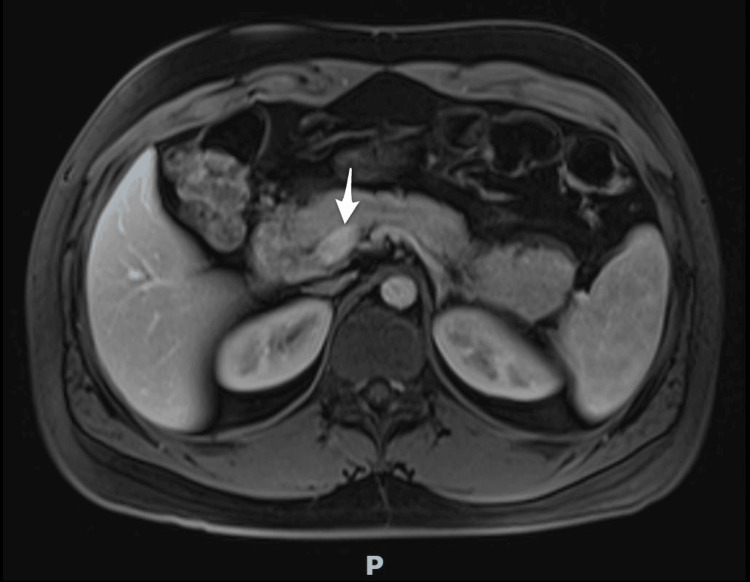
Delayed post-contrast axial pancreatic MRI demonstrating progressive enhancement of the pancreatic body lesion (white arrow). Progressive delayed enhancement is a recognized imaging feature of autoimmune pancreatitis and supports an inflammatory rather than malignant etiology.

Following multidisciplinary discussion, treatment with oral prednisolone (40 mg/day) was initiated based on the high clinical and radiological suspicion of AIP. The patient had not received intravenous fluid resuscitation prior to the initial CT examination.

At the two-week follow-up visit, the patient was asymptomatic and demonstrated marked clinical improvement, with good tolerance to corticosteroid therapy and no clinically relevant adverse effects. Prednisolone 40 mg/day was maintained for two weeks, followed by a tapering regimen of 5 mg per week. The planned total duration of corticosteroid therapy was six months.

However, given the absence of histological confirmation at treatment initiation and the potential impact of corticosteroids on subsequent tissue characterization, repeat pancreatic MRI and endoscopic ultrasound (EUS), with consideration of tissue acquisition, were requested to further support the diagnosis and definitively exclude alternative etiologies, particularly pancreatic malignancy. No follow-up imaging had been performed at the time of the first clinical reassessment.

## Discussion

AIP remains one of the most challenging benign conditions in the differential diagnosis of pancreatic masses, as its clinical and radiological presentation may closely resemble pancreatic malignancy [1,2,4,8]. This overlap frequently leads to extensive oncological investigation and, in selected cases, unnecessary invasive procedures or surgical intervention [2,4].

The present case illustrates a clinically relevant diagnostic dilemma frequently encountered in practice: balancing the pursuit of histological confirmation against the potential risks of delaying treatment in a potentially steroid-responsive disease.

Several findings collectively increased diagnostic confidence for AIP. From a radiological perspective, MRI demonstrated multifocal pancreatic involvement with progressive delayed enhancement, preservation of the main pancreatic duct calibre, and absence of significant biliary obstruction-features that are atypical for pancreatic adenocarcinoma and more suggestive of an inflammatory process. Typical imaging findings of AIP include long-segment (>1/3 of the duct length) or multifocal narrowing of the main pancreatic duct, absence of significant upstream ductal dilatation, and, in some cases, associated bile duct stenosis [1,2,4]. Multifocal pancreatic disease itself represents an uncommon pattern of presentation and may further complicate differentiation from neoplastic lesions [4,5].

The clinical presentation also supported the diagnosis. Type 2 AIP typically affects younger patients and frequently presents with recurrent episodes of acute pancreatitis, in contrast to type 1 disease, which more commonly manifests with painless obstructive jaundice and systemic IgG4-related disease [2-4]. In our patient, recurrent emergency department presentations for persistent abdominal pain and imaging evidence of pancreatic inflammation were consistent with this pattern.

Serological evaluation further supported an immune-mediated process, revealing elevated total IgG, mildly increased IgG4 concentrations, and positive ANA. Although elevated IgG4 is classically associated with type 1 AIP and IgG4-related disease, mild elevations may occasionally occur in patients with imaging characteristics favoring type 2 disease and should therefore not be interpreted in isolation. Importantly, mildly elevated IgG4 levels have also been reported in a subset of patients with pancreatic adenocarcinoma, further emphasizing the limited specificity of this biomarker when considered independently of clinical and radiological findings [2-5].

Importantly, alternative causes of pancreatitis were systematically excluded. The patient had no evidence of biliary disease, hypertriglyceridaemia, hypercalcaemia, significant alcohol exposure, or biochemical and radiological findings supporting pancreatic malignancy.

According to the International Consensus Diagnostic Criteria (ICDC), diagnosis of AIP relies on integration of pancreatic imaging, serological findings, histopathology, involvement of other organs, and response to corticosteroid therapy [1]. In the present case, although several diagnostic domains supported AIP, definitive classification remained limited by the absence of histopathological confirmation. While the patient’s age and MRI pattern favored type 2 AIP, the presence of mildly elevated IgG4 levels precluded confident subtype allocation. Accordingly, the most appropriate designation was considered to be probable AIP with imaging features favoring type 2 disease, rather than definitive type 2 AIP [1,5].

An additional layer of complexity arose from the initiation of corticosteroid therapy before endoscopic ultrasound-guided tissue acquisition. Although early treatment was considered clinically justified given the high pre-test probability of AIP and favorable clinical evolution, corticosteroid exposure may reduce subsequent diagnostic yield and complicate later histological interpretation [6,7,9].

At follow-up, the patient remained asymptomatic and demonstrated sustained clinical improvement throughout corticosteroid tapering, without clinically relevant adverse effects. Repeat pancreatic MRI and endoscopic ultrasound with consideration of tissue acquisition were requested to strengthen diagnostic certainty and definitively exclude alternative diagnoses, particularly pancreatic malignancy [10]. The prognosis of AIP is generally favorable, particularly in patients who respond promptly to corticosteroid therapy and do not present with obstructive jaundice, extensive biliary involvement, or evidence of chronic pancreatic insufficiency. In this context, our patient's rapid clinical response and absence of jaundice at presentation were considered reassuring prognostic features.

Overall, this case emphasizes the importance of integrating radiological, serological, and clinical findings when evaluating pancreatic mass-like lesions and reinforces that corticosteroid responsiveness should be interpreted as supportive evidence rather than a substitute for definitive diagnostic confirmation whenever feasible.

## Conclusions

AIP may closely mimic pancreatic malignancy, particularly when presenting as multifocal pancreatic masses. This case highlights the importance of integrating clinical, serological, and imaging findings to support diagnosis and guide management when histological confirmation is not immediately available. Careful multidisciplinary evaluation and structured follow-up remain essential, particularly when corticosteroid therapy is initiated before tissue diagnosis.
